# Dual-energy computed tomography as a lower radiation dose alternative to perfusion computed tomography in tumor viability assessment

**DOI:** 10.1038/s41598-022-27221-8

**Published:** 2023-01-04

**Authors:** Arkadiusz Zegadło, Aleksandra Różyk, Magdalena Żabicka, Ewa Więsik–Szewczyk, Artur Maliborski

**Affiliations:** 1 Department of Radiology, Military Institute of Medicine- National Research Institute, Szaserów 128, 04-141 Warsaw, Poland; 2 Department of Internal Medicine, Pulmonology, Allergy and Clinical Immunology, Central Clinical Hospital of the Ministry of National Defense, Military Institute of Medicine-National Research Institute, Warsaw, Poland

**Keywords:** Cancer, Cancer, Respiratory tract diseases

## Abstract

To present the utility of dual-energy computed tomography (DECT) in the assessment of angiogenesis of focal lesions as an example of a solitary pulmonary nodule (SPN). This prospective study comprised 28 patients with SPN who underwent DECT and perfusion computed tomography (CTP), according to a proprietary protocol. Two radiologists independently analyzed four perfusion parameters, namely blood flow (BF), blood volume (BV), the time to maximum of the tissue residue function (Tmax), permeability surface area product (PS) from CTP, in addition to the iodine concentration (IC) and normalized iodine concentration (NIC) of the SPN from DECT. We used the Pearson R correlation and interclass correlation coefficients (ICC_s_). Statistical significance was assumed at p < 0.05. The mean tumor size was 23.5 ± 6.5 mm. We observed good correlations between IC and BF (r = 0.78, p < 0.000) and NIC and BF (r = 0.71, p < 0.000) as well as between IC and BV (r = 0.73, p < 0.000) and NIC and BV (r = 0.73, p < 0.000) and poor correlation between IC and PS (r = 0.38, p = 0.044).There was no correlation between NIC and PS (r = 0.35, p = 0.064), IC content and Tmax (r = − 0.28, p = 0.147) and NIC and Tmax (r = − 0.21, p = 0.266). Inter-reader agreement on quantitative parameters at CTP (ICC_PS_ = 0.97, ICC_Tmax_ = 0.96, ICC_BV_ = 0.98, and ICC_BF_ = 0.99) and DECT (ICC_IC_ = 0.98) were excellent. The radiation dose was significantly lower in DECT than that in CTP (4.84 mSv vs. 9.07 mSv, respectively). DECT is useful for the functional assessment of oncological lesions with less exposure to radiation compared to perfusion computed tomography.

## Introduction

Of 14 million malignant tumors diagnosed worldwide in 2012, approximately 13% (1.8 million) were lung cancers. They are the most common malignant neoplastic disease in the men (16.7%) and the third most common disease in women (8.7%), following breast cancer and colorectal cancer^1^. Current multimodal treatment strategies utilizing induction chemotherapy or radiotherapy increase the number of patients undergoing a surgical resection of the tumor. They are extremely effective for patients with early stage lung cancer. The 5-year survival rate of patients with early surgical resection is 60–80%^2^. In contrast, patients with advanced lung cancer have an unfavorable prognosis, with a 5-year survival rate of 7–15%^2,3^. Computed tomography (CT) is often the first examination performed to detect lung tumors in imaging tests^4^.

Tumor angiogenesis is a key component of tumor diagnosis and treatment. Neovascularization-mediated tumor enhancement by >  + 20 Hounsfield units (HU)^5,6^ indicates malignancy, and is an important criterion for the differentiation of lesions^7–9^. The vascularization of pulmonary lesions is complex. Primary bronchial carcinomas are principally nourished by bronchial circulation; however, in several cases, the vascularization component of the pulmonary artery is present and grows for tumors around the periphery of the lung^10^. The degree of tumor vessel development, the degree of organization of their network, and the permeability to the tumor matrix determine the anti-angiogenic treatment strategy^11^.

Perfusion computed tomography (CTP) is a dynamic test that enables the in vivo assessment of the viability and degree of vascularization of the tumor, which affects the diagnosis, staging assessment, prognosis, and monitoring of the response to anti-angiogenic treatment^12–17^. Tumor microvessel density, as measured by the microvessel density (MVD) value, and vascular endothelial growth factor (VEGF) expression, as assessed by histopathology, are positively correlated with the results of the solitary pulmonary nodule (SPN) CTP examination. This confirms the significance of CTP as a specific biomarker in imaging for monitoring the effects of chemotherapy and radiotherapy^18,19^. CTP is a dynamic and volumetric examination that includes multiple scans of a designated area in a fixed time span. Therefore, it comprises the mean radiation dose of CTP for chest 1.5 times higher than the dose of the routinely used CT protocol for chest examination (1288.8 mGy cm vs. 885.2 mGy cm, respectively)^20^.

Dual-energy CT (DECT) examination enables the quantification of iodine concentration in selected regions of interest (ROI), thereby indirectly indicating the degree of tumor vascularization. It is based on images dependent on the distribution of iodine in the examined tissues. Previous studies have reported on the usefulness of assessing images dependent on the concentration of iodine in DECT, compared to perfusion images^21–23^.

We aimed to evaluate the possibility of using a single DECT scan as an alternative to high-dose CTP for assessing solid lung lesion angiogenesis, which we believe could be particularly beneficial for oncology patients, who often require multiple periodic follow-up CT scans.

## Materials and methods

This prospective study was approved by the Bioethics Committee of the Military Institute of Medicine in Warsaw, Poland on January 22, 2020 (protocol code 1/WIM/2020) and was conducted in accordance with the Declaration of Helsinki. All patients were informed about the conditions of participation in the study and provided their written informed consent.

### Patients

**Figure 1:**
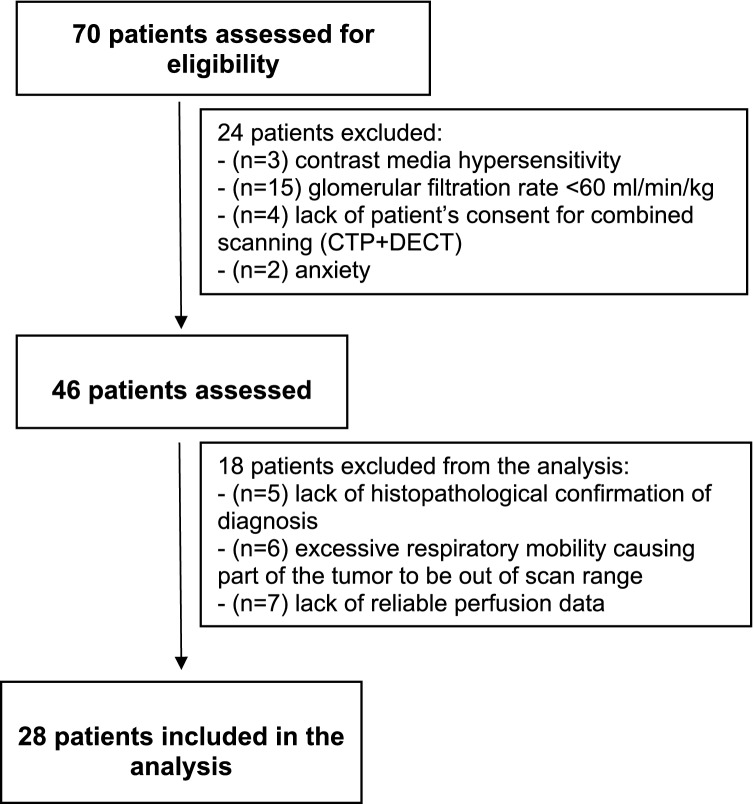
Inclusion and exclusion criteria for patient selection.

Between February 2020 and December 2020, we enrolled 70 patients aged ≤ 80 years who were hospitalized for further diagnosis and the treatment of a lung lesion, with a longitudinal size ≤ 30 mm. Patients with contrast medium hypersensitivity (n = 3), pregnancy (n = 0), glomerular filtration rate (GFR) < 60 ml/min/kg (n = 15), the refusal to undergo a double CT scan in the combined protocol (CTP + DECT) (n = 4), and anxiety responses (n = 2) were excluded from the study. Eventually, 46 patients underwent combined scanning (CTP + DECT) in accordance with our protocol. Of these patients, 18 were excluded from the analysis because of excessive respiratory mobility causing a part of the tumor to be moved out of the scan range (n = 6), the lack of reliable perfusion data (n = 7), and the lack of definitive histopathological diagnosis (n = 5). Eventually, 28 patients were included in the analysis (Fig. 1).

### CT protocol

All patients underwent a dynamic and polychromatic perfusion examination of the lung tumor. Five minutes later, the DECT examination was performed according to a self-designed protocol. The DECT examination were performed using a single-source DECT scanner with rapid kVp switching Discovery CT 750 HD (GE Healthcare, WI, USA). Before scanning, the subjects were instructed on the breathing method necessary to reduce motion blurring artifacts.

First, to determine the position of the SPN, we performed a native, helical chest scan, including diaphragm domes according to the following parameters: tube voltage, 120 kVp; current time product, 630 mAs; slice thickness 2.5 mm, and scanner rotation time 0.6 s.

**Figure 2:**
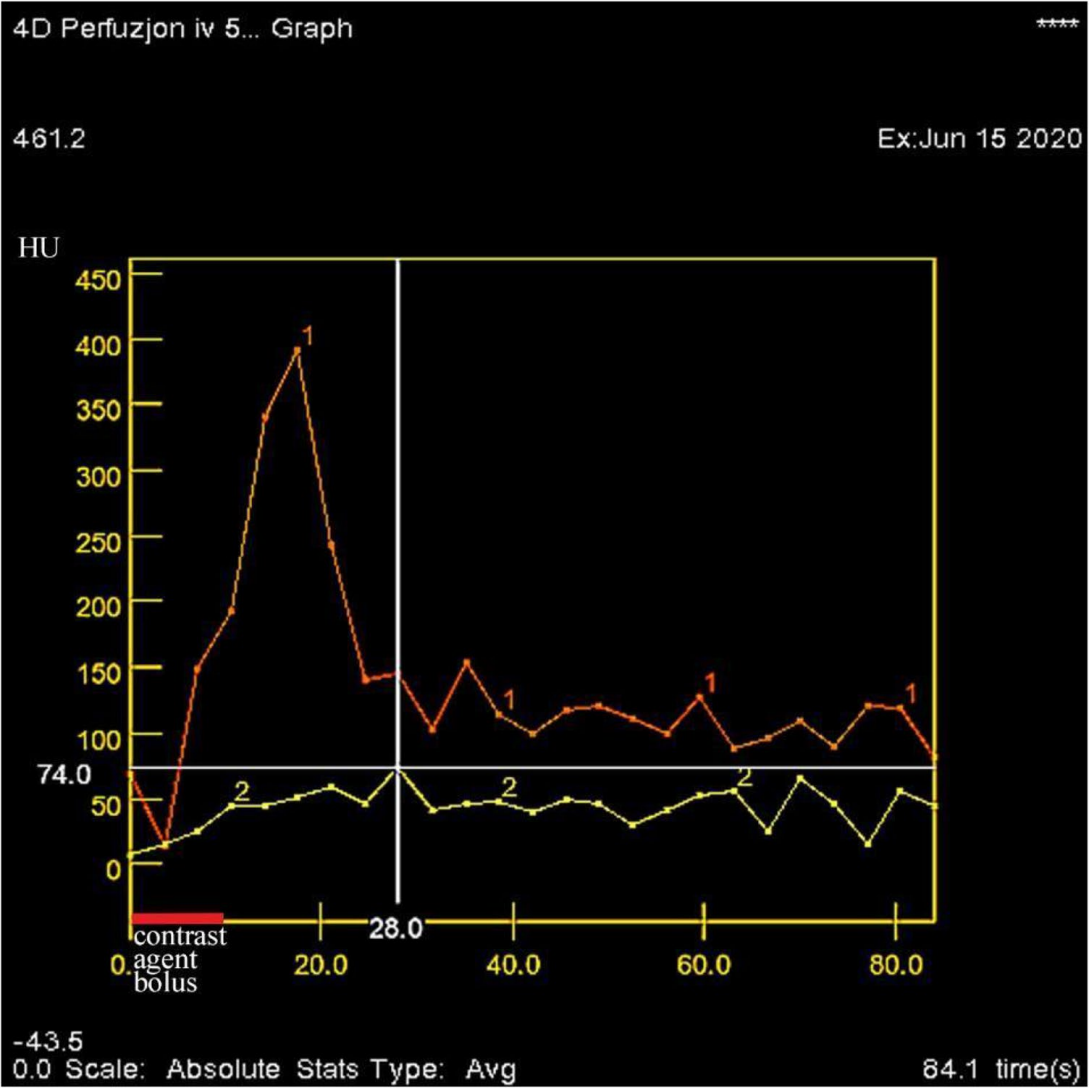
An example of CTP scanning*.* The contrast enhancement of the aorta is marked in red and the flow in the SPN is marked in yellow. The contrast bolus lasts for 10 s and begins simultaneously with the commencement of the scan. The greatest contrast enhancement of the tumor to + 74 HU is observed 28 s after contrast administration. *CTP* perfusion computed tomography.

After determining the tumor location, we performed a dynamic, axial CTP examination with a length of 40 mm (z-axis), which covered the area of the lesion with an unmoved scanner table according to the following protocol: tube voltage 80 kVp, current time product 100 mAs, large field of view (SFOV) 50 cm, slice thickness 5.0 mm and scanner rotation time 2 s. A bolus of 50 ml of non-ionic iodine contrast (400 mg/ml Iomeron, Bracco Imaging Deutschland GmbH, Konstanz, Germany) was used for CTP from an access through the cephalic vein at a rate of 5 ml/s, by injecting it with 20 ml of saline solution at a similar speed. In CTP, 30 scans were captured during a 60 s exposure at 2 s intervals. The beginning of the contrast agent bolus and the onset of CTP were simultaneous. The end of the contrast bolus was at the 10th second of the perfusion scan (Fig. 2).

The DECT phase was delayed by 300 s after the CTP phase. Moreover, it covered the similar tumor range according to the following protocol: tube voltage 70 kVe (range 80–140 kVp); tube current ~ 260 mA (modulated); scan time per rotation 0.8 s, large field of view (SFOV) 50 cm; slice thickness 1.25 mm; and detector collimation 64 × 0.625 mm. DECT involved the iodine contrast similar to that used in CTP at different doses depending on the body weight at a rate of 5 ml/s, injected by a bolus of 20 ml of saline solution. We used the following formula:

$$\mathrm{The \; amount \; of \; contrast i}.\mathrm{v}.= [(2 \; \mathrm{ ml }/ 1 \; \mathrm{ kg})- 50\; \mathrm{ ml}].$$

**Figure 3:**
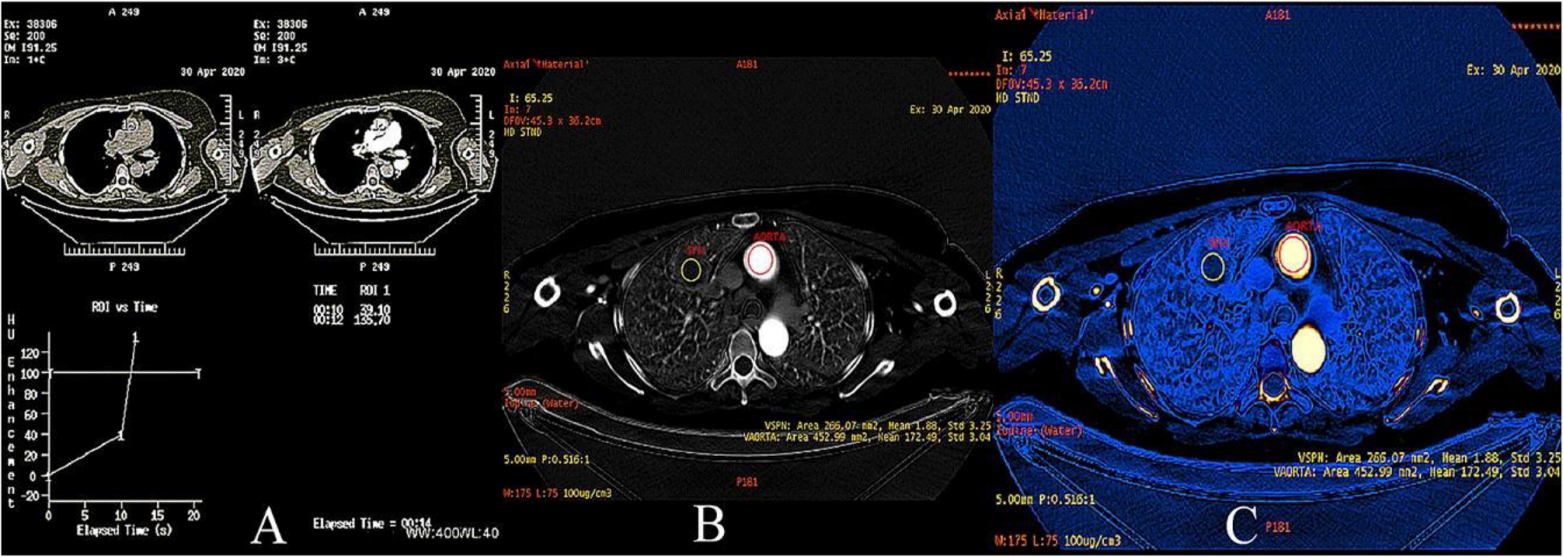
Exceeding the density of 100 HU in the ROI in the ascending aorta initiates the DECT (**A**) scan*.* Presentation of iodine concentrations at the reference site on the Linear Gray map (**B**) scan and the Color map (**C**) scan. DECT (ascending aorta—red ROI) and in the lung tumor (SPN—yellow ROI). Iodine concentration in the aorta and lung focal lesion is 172.49 × 100 µg/cm^3^ and 1.88 × 100 µg/cm^3^, respectively. *DECT* dual-energy computed tomography, *SPN* solitary pulmonary nodule, *ROI* region of interest.

The scanning was initiated when the concentration of the contrast agent in the thoracic aorta exceeded 100 HU (Fig. 3).

The images were reconstructed to a thickness of 5 mm for comparison with CTP images (Table 1).

**Table 1:** Acquisition protocol for dynamic CTP and DECT.

Phase	Tube potential	Tube current (mAs)	Rotation time (s)	Detector coverage (mm)	Slice thickness (mm)	Pitch/speed (mm/rot)	DLP (mGy-cm)	Total exposure time (s)	CTDI_VOL_ (mGy)	Number of acquisitions	Matrix size (mm)	DFOV
I Native	120 kVp	630	0.6	40	2.5	1.375:1/55.00	–	5.18	–	Helical	512 × 512	Large (50 cm)
**Contrast agent: 50 ml (flow 5 ml/s, bolus time 10 s)**												
II CTP	80 kVp	100	2.0	40	5.0	–	648,61	60.0	162,15	Axial 30	512 × 512	Large (50 cm)
**Contrast agent: [2 ml/kg–50 ml], (flow 5 ml/s); bolus tracking ROI: descending aorta, trigger threshold 100 HU**												
III DECT	70 keV	~ 260	0.8	40	1.25	0.516:1/20.62	346,41	6.83	19,67	Helical 109	512 × 512	Large (50 cm)

*DECT* dual-energy computed tomography, *CTP* perfusion computed tomography, *CTDI*_*vol*_ volume dose index, *DFOV* display field of view.

The radiation doses absorbed separately from dynamic CTP and DECT scanning were reported as two parameters, namely the dose length product (DLP, mGy-cm) and volume dose index (CTDIvol, mGy). The effective dose was calculated by multiplying the DLP with the conversation factor 0.0014 mSv/mGy^−1^ cm^−1^.

### Perfusion CT and DECT quantitative analysis

**Figure 4:**
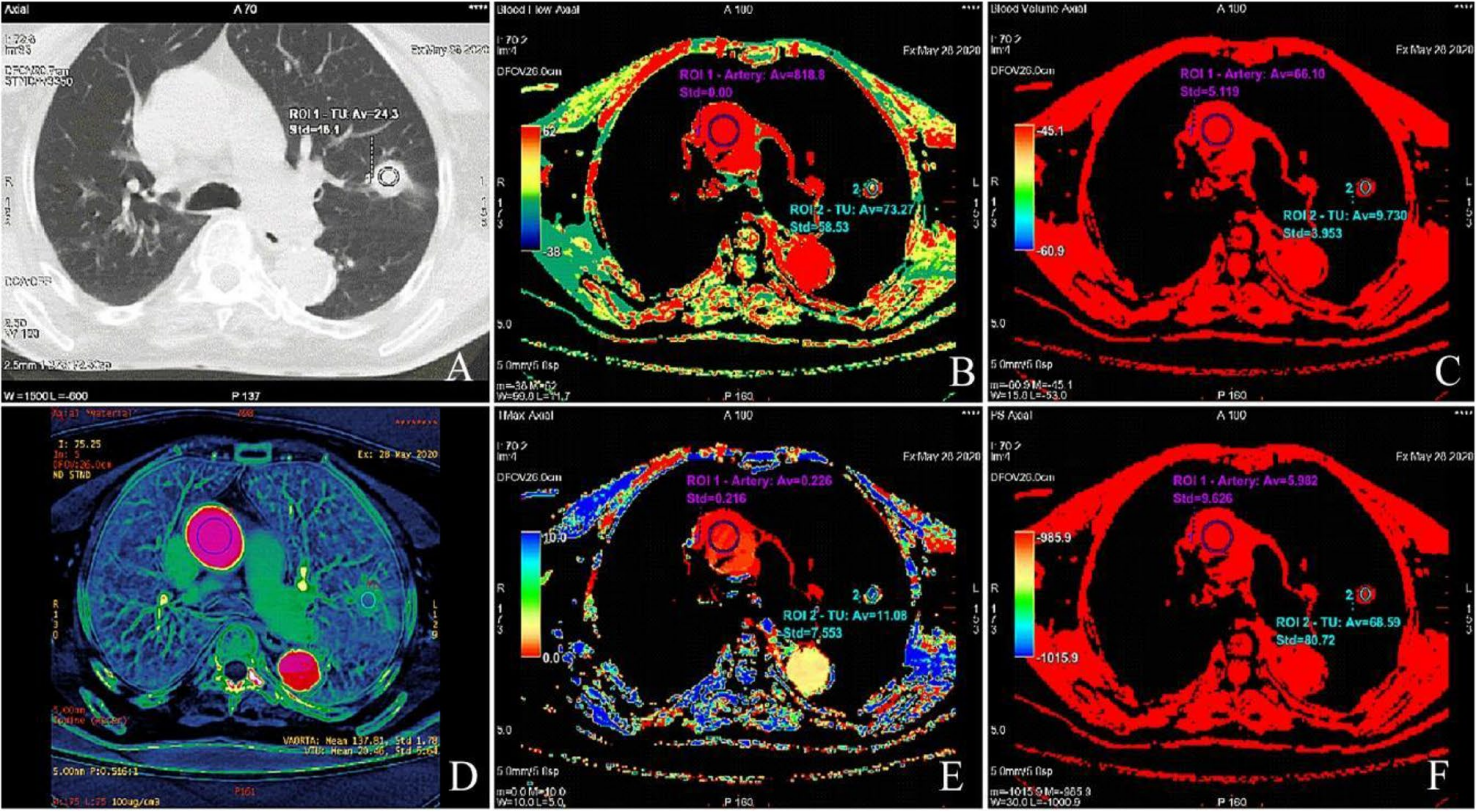
(**A**) CT scan displaying a circular ROI manually placed on the TU with average value of 24.3 HU. (**B**) BF 73.27 ml/100 g/min presents the mean flow rate through vasculature in the ROI tissue region of the TU. ROI 1 (purple color) represents the aorta, ROI 2 (green) denotes the tumor cross-section. (**C**) BV 9.73 ml/100 g presents the mean volume of flowing blood within a vasculature of the tissue region. (**D**) Contrast enhancement DECT reconstruction performed with Color maps French software on GE healthcare Workstation 4.7. (**E**) Tmax 11.08 s represents the delayed arrival of contrast bolus. (**F**) PS 68.59 ml/100 g/min denotes the total flux from the plasma to the interstitial space. *CTP* perfusion computed tomography, *ROI* region of interest, *TU* tumor, *BV* blood volume, *BF* blood flow, *Tmax* the time to the maximum of the residue function.

All combined CTP and DECT studies were sent from the scanner to the commercially available Advantage Workstation 4.7 (GE Healthcare). We analyzed the CTP images using the software CT perfusion 4D, GE Healthcare, based on the deconvolution method^24^. The studies were independently assessed by two radiologists AZ and MŻ with 17 and 27 years of experience in imaging lung pathology, respectively. The software automatically calculated the four perfusion parameters as follows: blood flow (BF) (ml/100 g/min), blood volume (BV) (ml/100 g), time-to-maximum of the tissue residue function (Tmax) (s), and permeability surface area product (PS) (ml/100 g/min) (Fig. 4).

Same radiologists performed the DECT examinations according to the Gemstone Spectral Imaging (GSI) General protocol in the GE Healthcare software Advantage Workstation 4.7. ROI measurement locations were manually based on similar areas marked independently in CTP measurements, and were performed with the utmost care by both researchers. The results (× 100 µg/cm^3^) were read automatically, and displayed mean concentrations of the contrast agent iodine (IC) in the ROI regions corresponding to the CTP measurement sites.

### Statistical analyses

We used a commercial statistical software package (Statistica w 13.3 Windows, TIBCO Software Inc., Palo Alto, USA) for statistical analyses. Continuous variables are presented as mean ± standard deviation (SD), median, and 95% confidence interval (CI). The variables were assessed for normal distribution using the Kolmogorow–Smirnov test. We used the Pearson product–moment correlation coefficient (r) to compare the iodine concentration values and four perfusion parameters (BF, BV, Tmax, and PS) in SPNs. Interclass correlation coefficients (ICC) were assessed using the intra-reader agreement for estimating perfusion for each pair of variables. In order to minimize variation between patients, the nodule iodine concentration was normalized to IC in the aorta to derive a normalized iodine concentration (NIC) by the formula: $NIC=ICtumor:ICaorta$

Differences were considered statistically significant at p < 0.05.

## Results

### Patients and tumor characteristics

The 28 included patients comprised 15 women (mean age: 70 ± 10 years) and 13 men (mean age: 68.5 ± 6 years). All patients had a single lung tumor that was assessed on CTP and DECT as well as examined histopathologically. Of these tumors, 10 (36%) were adenocarcinomas, seven (25%) were squamous cell carcinoma, four (14%) were hamartomas, three were granulomas (11%), and one each (4%) comprised carcinoid tumor, large cell neuroendocrine carcinoma, pneumoconiosis, and diagnosed with hypersensitivity pneumonia (Table 2).

**Table 2:** Characteristics of the patients, localization, and the number solid lung nodules.

	N (%)	Age ± SD (year)	Lung	Lobe (N)	Diagnosis (N)
Total	28 100%	69 ± 9	Right	Upper 14	Adenocarcinoma 6 Granuloma 2 Squamous cell carcinoma 3 Hamartoma 2 Pneumoconiosis 1
				Middle 3	Squamous cell carcinoma 1 Carcinoid tumor 1 Hypersensitivity pneumonia 1
				Lower 5	Adenocarcinoma 2 Granuloma 1 Squamous cell carcinoma 1 Hamartoma 1
			Left	Upper 4	Adenocarcinoma 1 Squamous cell carcinoma 1 Hamartoma 1 Large cell neuroendocrine carcinoma 1
				Lower 2	Adenocarcinoma 1 Squamous cell carcinoma 1
Female	15 54%	70 ± 10	Right	Upper 6	Adenocarcinoma 3 Squamous cell carcinoma 1 Hamartoma 2
				Middle 1	Hypersensitivity pneumonia 1
				Lower 3	Adenocarcinoma 2 Granuloma 1
			Left	Upper 3	Adenocarcinoma 1 Hamartoma 1 Large cell neuroendocrine carcinoma 1
				Lower 2	Adenocarcinoma 1 Squamous cell carcinoma 1
Male	13 46%	68.5 ± 6	Right	Upper 8	Adenocarcinoma 3 Granuloma 2 Squamous cell carcinoma 2 Pneumoconiosis 1
				Middle 2	Squamous cell carcinoma 1 Carcinoid tumor 1
				Lower 2	Squamous cell carcinoma 1 Hamartoma 1
			Left	Upper 1	Squamous cell carcinoma 1
				Lower 0	0

*N* number, *y* years, *SD* standard deviation.

The mean tumor size was 23.5 ± 6.5 mm (median 26 mm, CI 5.3–9.0 mm), with a native density of + 20.5 ± 7.7 HU (median + 20.5 HU, CI 5.9–10.2 HU). The mean SPN enhancement following contrast administration was 49.4 ± 21.9 HU (median 51.9 HU, CI 17.3–29.8 HU).

### CTP and DECT quantitative analysis

Two radiologists recorded two measurements of each of the four CTP parameters as follows: PS, Tmax, BV, BF, and IC. Interreader agreement for the quantitative parameters on perfusion CT (ICC_PS_ = 0.97, ICC_Tmax_ = 0.96, ICC_BV_ = 0.98, and ICC_BF_ = 0.99) and DECT (ICC_IC_ = 0.98) were excellent (Koo and Li, 2016). Table 3 summarizes the quantitative parameters from DECT and CTP.

**Table 3:** Quantitative parameters from perfusion computed tomography and dual-energy computed tomography.

Perfusion CT	Mean ± SD	Median	CI (− 95%–+ 95%)
PS (ml/100 g/min)	28.4 ± 26.3	28.1	18.2–38.6
Tmax (s)	6.1 ± 4.6	5.4	4.2–7.9
BF (ml/100 g/min)	133.1 ± 83.7	124.7	100.7–165.6
BV (ml/100 g)	6.9 ± 5.4	5.6	4.8–9.0
**DECT**			
IC (× 100 µg/cm^3^)	16.2 ± 8.7	16.3	12.8–19.6

*BF* blood flow, *BV* blood volume, *PS* permeability surface area product, *Tmax* the time-to-maximum of the tissue residue function, *SD* standard deviation, *CI* confidence interval.

### Correlations between perfusion CT (CTP) and dual-energy CT (DECT)

**Figure 5:**
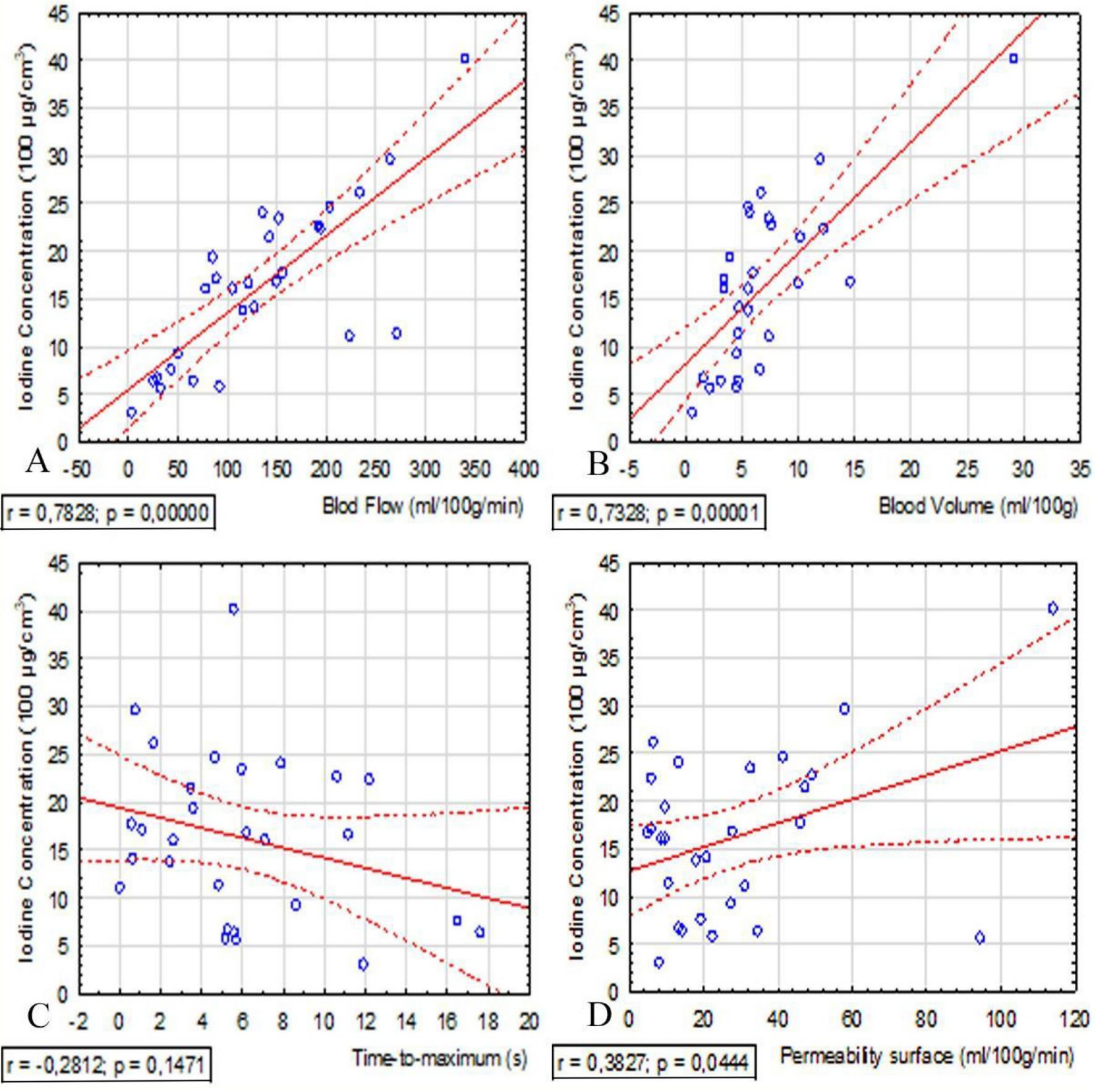
Scatterplot displaying Pearson correlation between the Blood Flow (**A**), the Blood Volume (**B**), the Time to maximum of the residue function (**C**), the Permability surface (**D**) at CTP and iodine concentration at DECT in SPNs with upper and lower lines indicating 95% confidence intervals. SPNs, *SPN* solitary pulmonary nodule, *r* correlation coefficient, *p* statistical significance.

We observed positive correlations between the concentration of iodine in the lung tumor and perfusion parameters^25^ as follows: strong correlation for BF (r = 0.78, p < 0.001), moderate correlation for BV (r = 0.73, p < 0.001) and poor correlation between the PS values (r = 0.39, p = 0.044). We did not observe a statistically significant relationship between the iodine content in the lung tumor and Tmax (r = − 0.28, p = 0.147) (Fig. 5, Table 4).

**Table 4:** Correlations of quantitative parameters between perfusion computed tomography and dual-energy computed tomography in patients with solitary pulmonary nodule (SPN).

Correlation	r	r^2^	p
BF and IC	+ 0.78	0.61	0.000
BV and IC	+ 0.73	0.53	0.000
PS and IC	+ 0.38	0.14	0.044
Tmax and IC	− 0.28	0.08	0.147
BF and NIC	+ 0.71	0.53	0.000
BV and NIC	+ 0.73	0.53	0.000
PS and NIC	+ 0.35	0.12	0.064
Tmax and NIC	− 0.21	0.05	0.266

*BF* blood flow, *BV* blood volume, *PS* permeability surface area product, *Tmax* time-to-maximum of the tissue residue function, *IC* iodine concentration, *NIC* normalized iodine concentration: R, correlation coefficient; R^2^, square of the correlation coefficient; and P, statistical significance.

Correlations between perfusion and parameter normalized IC in lung tumors were as follows: BF (r = 0.71, p < 0.000), moderate correlation for BV (r = 0.73, p < 0.000). We did not observe a statistically significant relationship between the NIC and PS values (r = 0.35, p = 0.064) and Tmax (r = − 0.21, p = 0.266). Correlation between NIC and IC was r = 0.83, p = 0.000.

### Radiation dose

A fixed scanning protocol was used in this study. Therefore, the volumetric CT dose rate (CTDIvol) was a constant value of 162.15 mGy for each dynamic CTP scan (1 scan/2 s/60 s). However, for a single DECT scan, the dose was much lower at 19.67 mGy. Simultaneously, DLP measurements for CTP and DECT scans were 648.61 mGy-cm (9.07 mSv) and 346.41 mGy-cm (4.84 mSv), respectively (the effective dose was calculated by multiplying the DLP with the conversation factor 0.0014 mSv/mGy^−1^ cm^−1^).

## Discussion

The DECT modality displays the accumulation of a contrast agent measured in conventional CT in HU units as its concentration in 1 cm^3^ of tissue, which is a unique solution. In pulmonary diagnostics it is used to assess malignancy of lung tumors^26,27^ or to differentiate the subtypes of malignant tumors^28^, assess lung vessels^29–31^ and lung tissue pathologies^32,33^.

Our findings demonstrated a strong correlation between the iodine content measured on IC maps and normalized IC maps in the single source modality, fast kV-switching DECT and perfusion parameters BF (R = 0.78 for IC and R = 0.71 for normalized IC) and BV (R = 0.73 for IC and R = 0.73 for normalized IC) in the vascular bed of a lung tumor 23.5 ± 6.5 mm of size, with comparatively lower exposure to the biological effects of radiation. Furthermore, the degree of this relationship demonstrated a high level of statistical significance (p < 0.000). The similar results of BV in comparison to IC and normalized IC indicate that this parameter is less dependent on the method of measuring the iodine concentration on DECT maps.

This was consistent with the results of studies on hepatocellular carcinoma by Mulé et al.^34^. In 2018, other authors^35^ demonstrated a correlation between IC and BF measurements during the assessment of the pancreas in healthy participants and patients with cancer. In a study on colorectal cancers^23^, Kang et al. indicated a relationship between perfusion parameters and the concentration of iodine in the tumor vessels with lower radiation exposure of DECT, compared to CTP. Strong correlation between the concentration of iodine on DECT maps in the arterial phase of contrast enhancement and perfusion in the hepatocellular carcinoma area^22^ is another example of the multidisciplinary application of DECT in the dynamic assessment of organ lesions as an alternative to PCT. Zhu et al.^36^ used this two modalities to differentiate the benign and malignant pulmonary nodules and showed that the iodine parameters from DECT significantly correlated with BV and BF, but with lower sensitivity and specificity for IC (86, 67 and 72, 73) in DECT in comparison to BV (94, 44 and 73, 33).

Numerous authors described a correlation of both BF and BV with the biomarkers of angiogenesis, namely microvessel density (MVD) and vascular endothelial growth factor (VEGF) concentration. BV correlates with the number of vessels that display the volume of blood collected in them^37^. The increase in tumor BF is an effect of the increase in blood flow at the arteriovenous connections that nourish the growing tumor, which translates into an assessment of tumor viability. The scale of reports appears promising and indicates the usefulness of CTP as a non-invasive technique to assess tumor viability^12–14,38^ and indicates the utility of CTP in differentiating between benign and malignant SPN lesions^39^. Moreover, it is reportedly useful in the histological differentiation between adenocarcinoma (AC) and squamous cell carcinoma, thereby indicating greater benefits of anti-angiogenic treatment in patients with AC^40^. Researchers have also mentioned the usefulness of CTP as a predictor of efficacy and a biomarker of the effects of anti-angiogenic therapy in the treatment of sarcoma^18^, non-small cell lung cancer^41^, pancreatic cancer^42^ and colorectal cancer^43^.

However, CTP is a dynamic examination that requires multiple scans and is associated with a high dose of absorbed radiation. The expected dose in CTP with a satisfactory signal-to-noise ratio is a maximum of 20 mSv or 30 mSv for a tissue volume of 4 cm in or larger volumes, respectively^24^.

The as low as reasonably achievable (ALARA) rule recommends limiting the patient's exposure to ionizing radiation to the necessary level, such that the benefits of the study outweigh the risk of side effects. The low average dose of absorbed radiation denotes the potential of DECT modality in comparison to CTP in the context of ALARA principle, and was 4.84 mSv—meaning about 50% of CTP dose in the present study (9.07 mSv).

The differences in radiation dose between those two modalities could be much higher, even if the effective radiation dose of CTP is as low as possible. Zhu et al.^36^ using a low-dose CTP achieved the effective radiation dose 4.67 ± 0.26 mSv vs 0.32 ± 0.10 mSv in DECT. Moreover DECT in comparison to CTP is much less dependant on the maximum length of examination in z-axis and respiratory mobility during procedure.

The DECT modality could be included in the assessment of tumor staging, with the simultaneous assessment of the effectiveness of oncological treatment based on the use of IC maps instead of BV and BF. The latter correlate with the biomarkers of MVD and VEGF angiogenesis and indicate the degree of response to anti-angiogenic therapy in neoplastic diseases. A positive response to treatment was denoted by a decrease in both values, i.e., BV and BF in the CTP study. In contrast, in the arterial phase of DECT, it corresponded to a decrease in iodine concentration in the lesion^44^.

Our study had some significant limitations. First, we included a small sample size. Second, respiratory mobility during the dynamic 1-min CTP scan could be minimized, but not completely avoided. Additionally CTP in our 64-row scanner (a very common type of scanner in Polish hospitals) covered only 4 cm of length in z-axis. For those two reasons 13 patients of the study group were eliminated: 6 patients because of respiratory mobility and 7 patients because of lack of reliable perfusion data. Another restriction applied to different ROIs that were manually inserted. Therefore, they did not reflect identical places on the perfusion and DECT maps. Finally, lack of patients undergoing anti-angiogenic oncological treatment during the analysis was also a limitation. Despite these limitations, we obtained high reproducibility of the measurements as assessed by the ICC test. Notably, tumor heterogeneity is a natural limitation of both techniques.

## Conclusions

Our long-term goal was to use the DECT modality in the CT oncology protocol. We believe that using DECT could be particularly beneficial in assessing tumors that display an apparent growth that is in fact caused by e.g. hemorrhage associated with anti-angiogenic treatment. The Response Evaluation Criteria in Solid Tumors (RECIST 1.1) and modified RECIST 1.1 for immune-based therapeutics (iRECIST)^45^ are based on the measurement of the size of lesion. Using RECIST, such tumors would be described as progressing^46,47^, however DECT imaging could make it possible to differentiate such changes from actual tumor growth^48^. Such modification of the oncology protocol could have significant implications in further patient management.

In conclusion, the results of our study highlighted the usefulness of DECT in the assessment of functional changes in the tumor vascular bed. Although it requires further studies, DECT examination could possibly replace CTP in the future in the functional assessment of oncological changes during early diagnosis and monitoring the effects of treatment, with comparatively lower exposure to the effects of radiation.

## Data Availability

Datasets generated analysed during the current study are not publicly available due to personal data protections laws, however they can be made available from the author on a reasonable request (in anonymized form). In such cases, please contact dr Arkadiusz Zegadło (azegadlo@wim.mil.pl).

## Abbreviations

BF: Blood flow
BV: Blood volume
CTDI_vol_: Volume computed tomography dose index
CT: Computed tomography
CTP: Perfusion computed tomography
DECT: Dual Energy Computed Tomography
DLP: Dose length product
IC: Iodine concentration
NIC: Normalized iodine concentration
PS: Permeability surface area product
ROI: Region of interest
SPN: Solitary pulmonary nodule
Tmax: Time-to-maximum of the tissue residue function
TU: Tumor
VEGF: Vascular endothelial growth factor
